# Impact of TauroLock^™^-HEP500 versus unfractionated heparin for prevention of catheter complications in children with malignancy: a prospective, randomized, controlled study

**DOI:** 10.1186/s40001-025-03413-6

**Published:** 2025-11-18

**Authors:** Aziz Eghbali, Arya Shirani, Mobin Obeidinia, Makan Ziafati, Ali Ghasemi, Kazem Ghaffari

**Affiliations:** 1 Clinical Research Development Center of Aliasghar Hospital, Iran University of Medical Sciences, Tehran, Iran; 2 Research Scholar, School of Medicine, Iran University of Medical Sciences, Tehran, Iran; 3 Abnormal Uterine Bleeding Research Center, Semnan University of Medical Sciences, Semnan, Iran; 4 Cancer Research Center, Semnan University of Medical Sciences, Semnan, Iran; 5 Department of Biochemistry and Hematology, Faculty of Medicine, Semnan University of Medical Sciences, Semnan, Iran; 6 Department of Hematology and Blood Transfusion Sciences, School of Allied Medical Sciences, Tehran University of Medical Sciences, Tehran, Iran; 7 Department of Basic and Laboratory Sciences, Khomein University of Medical Sciences, Khomein, Iran; 8 Student’s Committee Research Center, Khomein University of Medical Sciences, Khomein, Iran; 9 Student’s Scientific Research Center, Tehran University of Medical Sciences, Tehran, Iran

**Keywords:** Catheter dysfunction, CRBSI, Malignancy, Lock therapy, Heparin, Taurolidine

## Abstract

**Background and aims:**

Central venous catheters (CVCs) are essential for drug delivery in pediatric oncology patients but are associated with complications such as infection and thrombosis. This study aimed to compare the effects of taurolidine–citrate and unfractionated heparin lock solutions on catheter function, infection and thrombosis rates, and inflammatory markers in children with malignancies.

**Methods:**

In this randomized, controlled trial, 76 pediatric oncology patients were allocated to receive either TauroLock^™^-HEP500 (containing taurolidine, 4% citrate, and 500 IU/mL heparin) or standard unfractionated heparin as the catheter lock solution. Patients were followed for 6 months. Laboratory evaluations, including complete blood count (CBC), high-sensitivity *C*-reactive protein (hs-CRP), and interleukin-6 (IL-6), were performed at baseline, 1 month, and 6 months, or upon clinical suspicion of infection.

**Results:**

At 6 months, hs-CRP levels were significantly lower in the taurolidine–citrate group (2.1 ± 0.2 vs. 5.5 ± 2.2, *p* = 0.001), as was total WBC count (3792.1 ± 325.3 vs. 4994.5 ± 462.1, *p* = 0.028). IL-6 levels showed no statistically significant difference (9.2 ± 1.9 vs. 14.0 ± 3.1, *p* = 0.067). The incidence of catheter-related infections (HR 3.55, 95% CI 0.68–18.4, *p* = 0.460) and thrombosis (HR 4.13, 95% CI 0.43–39.91, *p* = 0.221) did not differ significantly between groups.

**Conclusion:**

Taurolidine–citrate exhibited a modest anti-inflammatory effect, reflected by reduced hs-CRP and WBC levels, without significant improvement in catheter-related complications or IL-6. The lack of major clinical benefit may relate to the heterogeneous and immunocompromised nature of pediatric oncology patients. Larger, adequately powered studies are warranted to clarify the long-term efficacy and safety of taurolidine–citrate in this population. Clinical Trials as IRCT20201107049296N4.

## Introduction

Central venous catheters (CVCs) are a standard route for drug administration in pediatric oncology patients receiving chemotherapy, despite some well-known complications [1–3]. Chemotherapy remains a cornerstone of childhood cancer treatment, markedly improving prognosis [4–13]. In these patients, CVCs are indispensable for medication delivery, blood sampling, and supportive therapies, reducing the need for repeated venipuncture and ensuring reliable vascular access [14, 15]. Different types of CVCs are available, such as single- and double-lumen Hickman–Broviac catheters, Pressure Activated Safety Valve (PASV) catheters, and totally implantable devices like Port-a-Cath (PORT). Among them, single-lumen catheters generally show lower complication rates [16]. Catheter-related bloodstream infections (CRBSIs) remain a major concern due to their association with treatment delays, prolonged hospitalization, and increased healthcare costs [17]. Traditionally, heparin has been used for catheter locking to prevent occlusion; however, it may promote biofilm formation, which can contribute to infection risk [18, 19]. To address this, antimicrobial lock solutions have been developed. Taurolidine, an antimicrobial agent combined with citrate or heparin, has demonstrated promising results in reducing microbial adhesion and biofilm formation without promoting antimicrobial resistance [1, 20, 21].

TauroLock^™^ is a lock solution containing 1.35% taurolidine and 4% citrate, designed to prevent catheter-related infections. The TauroLock^™^-HEP500 formulation additionally includes heparin (500 IU/mL) to maintain catheter patency. Despite encouraging data, the evidence supporting taurolidine-citrate-heparin solutions remains moderate, and some controversy persists regarding their effectiveness. While certain guidelines do not recommend routine use of anticoagulants in CVCs (except for hemodialysis), heparin-containing locks are still widely employed in pediatric oncology centers, including ours (Ali Asghar Children’s Hospital, Tehran, Iran), due to the high risk of occlusion and thrombosis [22–25].

Therefore, this study was conducted to provide further evidence on the use of TauroLock^™^-HEP500 in pediatric oncology patients, focusing on its inflammatory, infective, and functional effects, which have not yet been comprehensively evaluated in this population.

## Materials and methods

### Ethical approval

The study protocol was approved by the Ethics Committee of Iran University of Medical Sciences (Approval Code: IR.IUMS.REC.1402.598) and registered at the Iranian Registry of Clinical Trials (IRCT20201107049296N4). All procedures followed the principles of the Declaration of Helsinki. Written informed consent was obtained from parents or legal guardians for minors, and directly from patients above the age of consent. Participants and their families were informed about their right to withdraw at any time without affecting their clinical care.

### Patient selection and randomization

**Fig. 1:**
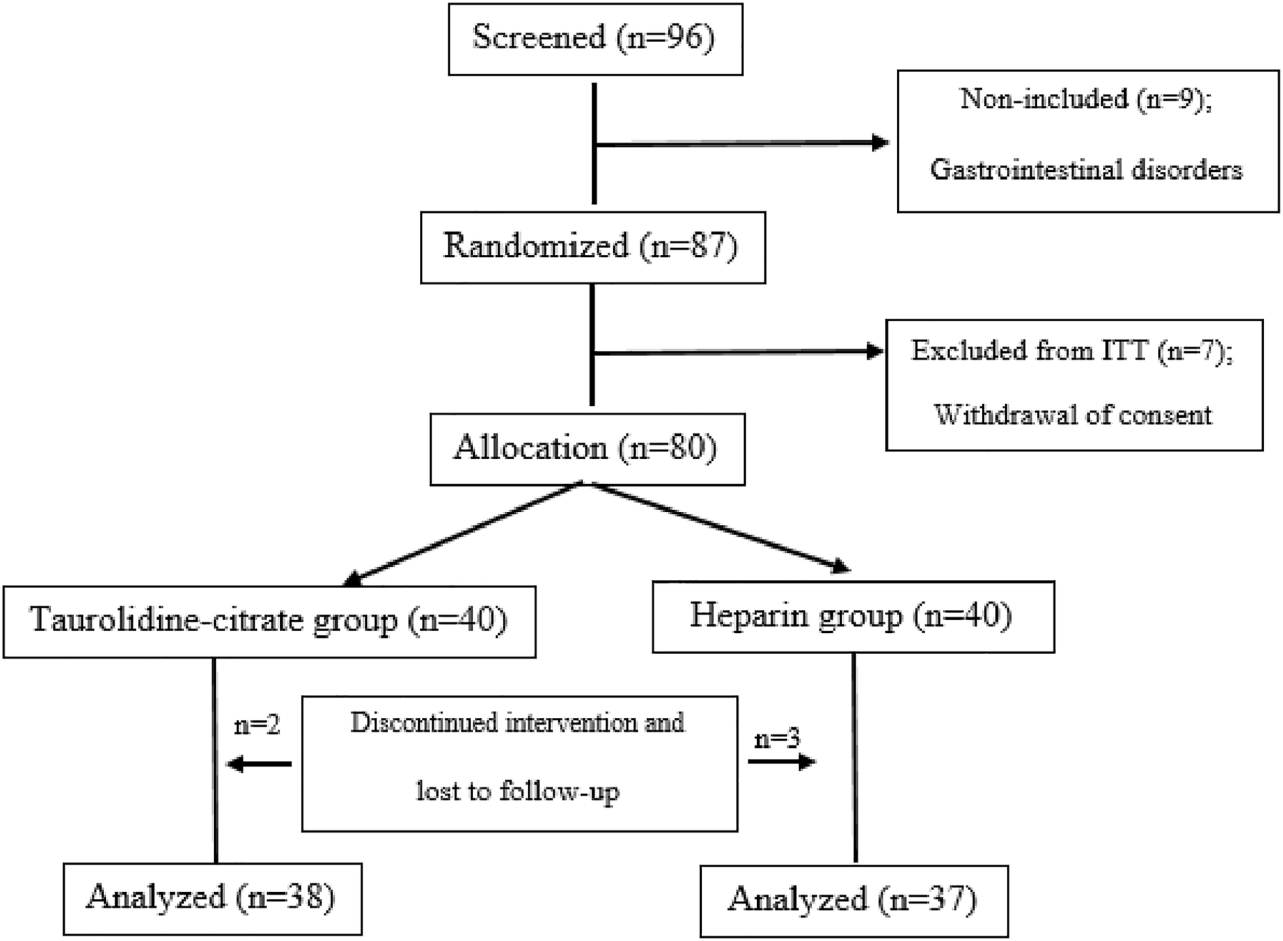
Flowchart of study procedure. ITT; intent-to-treat population

This randomized, open-label, controlled clinical trial enrolled 76 pediatric oncology patients (< 18 years) newly diagnosed with malignancies requiring chemotherapy at Ali Asghar Children’s Hospital (Tehran, Iran) between January and May 2024. The sample size was calculated based on the primary outcome (difference in catheter functional duration and IL-6 levels). Assuming a 25% baseline catheter dysfunction rate over 6 months and aiming for a 30% relative reduction (to 17.5%) with 80% power, *α* = 0.05, and a 10% dropout rate, 76 patients (38 per group) were required. Randomization was performed using a simple random number generator in Microsoft Excel. Each eligible patient received a unique ID, and random numbers were generated using the RAND function. IDs were sorted in ascending order according to the generated numbers, and patients were assigned to the taurolidine-citrate or heparin group in a 1:1 ratio. All patients received single-lumen, tunneled catheters (Polysite^®^, Vygon Medical Industries, UK) implanted subcutaneously by pediatric surgeons under sterile conditions. Catheters were used for chemotherapy and transfusions. The exit site was covered with a bio-occlusive dressing, cleaned with povidone–iodine, and redressed every 3 days. Catheters were locked after each chemotherapy cycle by a pediatric oncologist. Partial blinding was implemented: patients, laboratory personnel, and evaluators were blinded, but attending physicians and nurses performing the locking were not. A CONSORT flow diagram is presented in Fig. 1.

### Inclusion and exclusion criteria

Patients under 18 years of age with a confirmed diagnosis of malignancy, including leukemia, lymphoma, or solid tumors, who required the insertion of a tunneled CVC for chemotherapy or related supportive care, were eligible for inclusion. Only patients whose parents or legal guardians provided written informed consent were enrolled. At baseline, patients had to be free of any signs of active infection. Patients were excluded if they required specialized medical care outside the oncology department or were transferred to other units such as the intensive care unit or nephrology. Additional exclusion criteria included a history of coagulation disorders (e.g., hemophilia or von Willebrand disease), hematologic conditions not associated with neutropenia—such as sickle cell anemia or Diamond-Blackfan anemia—and known hypersensitivity to any components of the lock solutions, including taurolidine or heparin. Patients who were on systemic prophylactic antibiotics at the time of enrollment, those with poor prognosis and an expected survival of less than 3 months, or those who had experienced CRBSI or venous thrombosis within the past 3 months were also excluded from the study.

### Study design and interventions

After randomization, patients were divided into two groups: one group received 2.5–3 cc of taurolidine-citrate solution (taurolidine 1.35% and sodium citrate 4%, Taurolock^™^, Tauropharm, Waldbuttelburn, Germany) based on their clinical needs after each chemotherapy session. The other group received unfractionated heparin (5000 IU/mL; Sodium Heparin, Darou Pakhsh, Tehran, Iran). A volume of 0.2 mL (containing 1000 IU) was diluted with sterile normal saline to achieve a final concentration of 100 IU/mL, and the volume used to lock the catheter matched its internal lumen volume, typically ranging from 1 to 2 mL. The lock solution was aspirated without irrigation to deplete before starting the chemotherapy, and then the catheter was washed with 10.0 cc of N/S. After the secession was completed, irrigation with N/S was again performed, and the locking was performed as mentioned above. Participants, laboratory analysts, and outcome assessors were blinded to the group assignments, while clinicians responsible for catheter locking were not.

A pediatric oncologist professor handled the catheter using standard sterile techniques. During routine clinic visits, patients were examined for fever and other signs of infection. The type of pediatric malignancy was also noted in each group.

The catheter’s functional days were measured from the insertion date to the date of withdrawal due to dysfunction or cure of the malignancy.

Laboratory assessments, including complete blood count (CBC), high-sensitivity *C*-reactive protein (hs-CRP), and interleukin-6 (IL-6), were performed at baseline (time of catheter insertion) and up to 6 months later or earlier if clinical signs of infection appeared. The enzyme-linked immunosorbent assay (ELISA) method was used to measure serum CRP and IL-6 levels.

The primary endpoints of this study were serum IL-6 levels and the duration of catheter functionality. hs-CRP levels were considered a secondary outcome. In addition, although not predefined as primary or secondary endpoints, long-term catheter-related complications, including CRBSI and catheter thrombosis, were systematically recorded and analyzed, given their clinical importance.

If body temperature exceeded 38.5 °C for at least 4 h or there was one episode > 39 °C, a workup was conducted to identify the source of infection. When CRBSI was suspected, two blood cultures were obtained: one from peripheral blood and one from the central catheter. CRBSI diagnosis was based on differential time to positivity (DTP), defined as a growth detected at least 2 h earlier in the catheter sample compared to the peripheral sample [26]. To detect catheter-related thrombosis, Color Doppler ultrasonography was performed either at the 6-month follow-up or at the time of catheter removal.

### Statistical analysis

All statistical analyses were performed using SPSS version 27 (IBM Corp., Armonk, NY, USA). Continuous variables were presented as means ± standard deviation (SD) or medians (interquartile range [IQR]) depending on normality assessment, and categorical variables were expressed as frequencies and percentages. Chi-square or Fisher’s exact test was used for categorical data. Independent samples *t*-test or Mann–Whitney *U* test was used for continuous variables based on distribution. Cox proportional hazards regression was used to analyze time-to-event data, specifically to assess the time from catheter insertion to catheter removal due to any complication (e.g., infection, thrombosis, or malfunction). This method was chosen because it accounts for both the occurrence and timing of events, allowing for a more accurate estimation of the impact of lock solution type (taurolidine-citrate vs. heparin) on catheter survival. The model included treatment group as the main covariate, and hazard ratios (HRs) with 95% confidence intervals (Cis) were calculated to quantify the relative risk. All statistical tests were two-sided, and a *p*-value < 0.05 was considered statistically significant.

## Results

The primary outcomes of this trial were catheter functional duration and serum IL-6 levels. Secondary outcomes included hs-CRP, WBC, and the incidence of CRBSI, thrombosis, and catheter withdrawal.

### Catheter functionality duration

A total of 76 patients (42 males and 34 females) were enrolled and randomized into the taurolidine–citrate group (*n* = 38) and the heparin group (*n* = 38). Seventy-five patients completed the study. After a mean follow-up of 7.3 ± 2.3 months, 66 catheters remained functional, while 9 experienced dysfunction (3 infection-related, 3 due to infection and thrombosis, 1 thrombosis alone, and 2 mechanical failures). Catheter dysfunction occurred less frequently in the taurolidine–citrate group (3 events) compared with the heparin group (5 events), though the difference was not statistically significant (HR = 3.50; 95% CI 0.69–18.40; *p* = 0.131). Baseline demographic and clinical characteristics are summarized in Table 1.

**Table 1:** Characteristics of the study population

Variables	Taurolidine-citrate group (*N* = 38)	Heparin group (*N* = 37)	*p*
Age, years, mean ± SD	6.2 ± 3.8	6.1 ± 3.8	0.831
Sex, male n (%)	22 (58%)	20 (54%)	0.732
Type of malignancy;			
ALL	28 (73.6)	23 (62.1)	0.846
AML	2 (5.2)	4 (10.8)	
Neuroblastoma	2 (5.2)	3 (8.1)	
Wilms tumor	1 (2.6)	2 (5.4)	
Hepatoblastoma	1 (2.6)	2 (5.4)	
Osteosarcoma	1 (2.6)	1 (2.7)	
Rhabdomyosarcoma	0	1 (2.7)	
Ewing Sarcoma	0	1 (2.7)	
PNET	1 (2.6)	0	
Hodgkin lymphoma	1 (2.6)	0	
LCH	1 (2.6)	0	
hs-CRP;			
Baseline	2.7 ± 0.2	2.7 ± 0.3	0.582
1-month	2.6 ± 0.1	3.4 ± 0.6	0.780
6-month	2.1 ± 0.2	5.5 ± 2.2	**0.001**
IL-6;			
Baseline	7.1 ± 0.5	6.0 ± 0.4	0.231
1 month	7.0 ± 0.5	7.0 ± 0.6	0.943
6 months	9.2 ± 1.9	14.0 ± 3.1	0.067
Total WBC;			
Baseline	4376.3 ± 249.9	4648.6 ± 362.0	0.999
1 month	3771.0 ± 219.8	4267.5 ± 356.3	0.530
6 months	3792.1 ± 325.3	4994.5 ± 462.1	**0.028**

*N* number, *ALL* acute lymphoblastic leukemia, *AML* acute myeloid leukemia, *PNET* Pancreatic neuroendocrine tumors, *LCH* Langerhans cell histiocytosis, *hs-CRP* high-sensitivity CRP, *IL-6* Interlukin-6, *WBC* white blood cell count. Bold numbers indicate p < 0.05.

### Interleukin-6 (IL-6)

At baseline, IL-6 levels were slightly higher in the taurolidine–citrate group (8.6 ± 3.1 pg/mL) than in the heparin group (7.9 ± 2.8 pg/mL), but this difference was not significant (mean difference: 0.7 pg/mL; 95% CI − 0.5–1.9; *p* = 0.231). After 1 month, IL-6 levels remained similar between groups (taurolidine–citrate: 8.2 ± 3.4 pg/mL; heparin: 8.1 ± 3.2 pg/mL; 95% CI − 1.2–1.4; *p* = 0.943). At 6 months, IL-6 levels were slightly higher in the heparin group (9.8 ± 3.6 pg/mL) compared with the taurolidine–citrate group (8.3 ± 3.0 pg/mL), although this difference did not reach statistical significance (mean difference: 1.5 pg/mL; 95% CI − 3.1–0.1; *p* = 0.067).

### hs-CRP

At baseline, hs-CRP values were comparable between groups (taurolidine–citrate: 3.9 ± 1.8 mg/L; heparin: 4.1 ± 2.0 mg/L; mean difference: 0.2 mg/L; 95% CI − 0.9–0.5; *p* = 0.582). After 1 month, hs-CRP remained slightly higher in the heparin group (4.3 ± 2.1 mg/L) compared with the taurolidine–citrate group (4.0 ± 1.9 mg/L; 95% CI − 0.7–1.3; *p* = 0.780). However, at 6 months, hs-CRP was significantly higher in the heparin group (6.5 ± 2.4 mg/L) than in the taurolidine–citrate group (4.7 ± 2.0 mg/L; mean difference: 1.8 mg/L; 95% CI 0.8–2.9; *p* = 0.001).

### WBC

At baseline, WBC counts were slightly higher in the heparin group (7.1 ± 1.8 × 10⁹/L) than in the taurolidine–citrate group (7.0 ± 1.7 × 10⁹/L; 95% CI − 0.6–0.8; *p* > 0.999). After 1 month, the heparin group still showed higher WBC counts (7.3 ± 2.0 × 10⁹/L vs. 6.9 ± 1.6 × 10⁹/L; 95% CI − 0.4–1.2; *p* = 0.530). At 6 months, WBC counts were significantly elevated in the heparin group (8.2 ± 2.2 × 10⁹/L) compared with the taurolidine–citrate group (6.9 ± 1.9 × 10⁹/L; mean difference: 1.3 × 10⁹/L; 95% CI 0.2–2.4; *p* = 0.028).

### Outcome events

Eight patients had positive blood cultures: three in the taurolidine–citrate group and five in the heparin group. Six required catheter removal. Based on differential time to positivity (> 2 h), six cases were classified as catheter-related bloodstream infections (CRBSIs), while two (one per group) were considered colonizations. Infection rates did not differ significantly between groups (HR = 3.55; 95% CI 0.68–18.40; *p* = 0.460). Color Doppler ultrasonography detected thrombosis in four patients (three in the heparin group and one in the taurolidine–citrate group; HR = 4.13; 95% CI 0.43–39.91; *p* = 0.221). Catheter withdrawal occurred slightly more often in the heparin group (13.5%) than in the taurolidine–citrate group (10.5%), though this was not statistically significant (HR = 2.68; 95% CI 0.63–11.40; *p* = 0.181). Overall, positive blood cultures and Doppler-confirmed thrombosis were more frequent in the heparin group, though not statistically significant. All clinical and laboratory outcomes are summarized in Table 2. These secondary analyses complement the primary outcomes of catheter functionality and IL-6 levels, providing a comprehensive evaluation of both biochemical and clinical effects.

**Table 2:** Primary and secondary endpoints of the study

Outcome	Taurolidine-citrate group (*N* = 38)	Heparin group (*N* = 37)	*p*-value	HR [95% CI]
Main outcome; catheter withdrawal	4 (10.5)	5 (13.5)	0.181	2.68 [0.63–11.40]
Number of locks used (mean ± SD)	28.7 ± 7.5	29.1 ± 8.2	0.754	–
Total lock days (mean ± SD)	174.6 ± 23.2	170.3 ± 21.9	0.537	–
Components of Main Outcome;				
Infection	2 (5.3)	1 (2.7)	0.233	–
Thrombosis	1 (2.6)	0		
Infection and thrombosis	0	3 (8.1)		
Other	1 (2.6)	1 (2.7)		
Positive blood Culture	3 (8)	5 (13.5)	0.131	3.55 [0.68—18.40]
Thrombosis in Color Doppler Sonography	1 (2.6)	3 (8.1)	0.220	4.13 [0.43—39.91]

*N* number. *HR* Hazard Ratio, *CI* Confidence Intervals

## Discussion

Our findings suggest that taurolidine–citrate lock solutions in pediatric oncology patients may be associated with lower inflammatory marker levels (hs-CRP and WBC) after 6 months, indicating a modestly less pro-inflammatory profile compared to standard heparin locks. However, this biochemical improvement did not translate into significant clinical benefits regarding catheter functionality, thrombosis, or CRBSIs. No significant change was observed in IL-6, supporting the interpretation that taurolidine–citrate exerts minimal systemic immunomodulatory effects.

The absence of statistically significant clinical outcomes may reflect the limited sample size and exploratory nature of this preliminary study. Although the local antimicrobial properties of taurolidine are well established, its ability to prevent clinically evident CRBSI or thrombosis might be constrained in high-risk, immunocompromised pediatric oncology populations. The observed reduction in systemic inflammatory markers may therefore represent a general attenuation of inflammatory tone rather than a targeted anti-infective effect at the catheter site.

Previous studies have shown that patients with tunneled catheters often exhibit elevated serum CRP levels even in the absence of infection, suggesting chronic low-grade inflammatory activation [27]. Cytokines such as IL-6 are central to this process and contribute to vascular injury and endothelial dysfunction [28, 29]. Reported CRBSI frequencies vary widely depending on catheter type, population, and diagnostic criteria [30]. While international guidelines increasingly recommend normal saline alone for catheter locking, many pediatric oncology centers—including ours—continue to use heparin to maintain patency [23, 31]. However, heparin lacks antimicrobial activity, and supporting evidence for its efficacy remains limited.

A pooled meta-analysis of randomized controlled trials demonstrated that taurolidine-containing lock solutions significantly reduced infection rates compared with heparin, saline, or citrate alone (pooled IRR = 0.30; 95% CI 0.19–0.46), though study heterogeneity and risk of bias were considerable [32]. In pediatric oncology, few small trials have been published, with inconsistent results [1, 33–36]. For instance, the TAURCAT (Spain) and ATAPAC (France) studies found no significant reduction in CRBSI or catheter removal with taurolidine–citrate compared with heparin [37, 38], findings that align with our results. Similarly, our data showed a non-significant trend toward fewer catheter withdrawals in the taurolidine–citrate group. It is worth noting that prior pediatric studies often used CRBSI-related occlusion as the primary endpoint, whereas our trial evaluated overall catheter dysfunction—including occlusion, thrombosis, and mechanical failure—as the main functional outcome [33, 35].

Evidence from adult hemodialysis studies also supports taurolidine–citrate’s antimicrobial advantage, with substantially lower CRBSI rates (HR ≈ 0.29) compared with heparin, although catheter survival remained unchanged [39]. The discrepancy between these findings and ours may relate to differences in baseline conditions (end-stage renal disease vs. malignancy) and catheter dwell time. Meta-analyses have confirmed overall reductions in CRBSI risk with taurolidine–citrate but reported substantial heterogeneity (*I*^2^ = 55.6%) and limited pediatric data, consistent with our observations. Likewise, studies in parenteral-nutrition populations have shown reduced infection and occlusion rates [40, 41], but the patient populations differ considerably from ours.

To our knowledge, this is the first study to evaluate taurolidine–citrate’s effects on inflammatory markers—including hs-CRP, WBC, and IL-6—in pediatric oncology patients. Prior investigations in adult hemodialysis populations have yielded mixed results, with some reporting significant reductions in inflammatory biomarkers [21, 42], while others found no meaningful differences [43]. Such discrepancies likely reflect population heterogeneity, comorbidities, and lock formulation differences. Considering that systemic inflammation can influence chemotherapy tolerance and hematopoietic recovery [44], the potential immunomodulatory properties of taurolidine merit further investigation in this vulnerable group.

Regarding safety, previous studies have cited an unpleasant taste as the most common side effect of taurolidine–citrate [1, 29]. We observed no gastrointestinal or systemic adverse events in our cohort. Adherence to standard aspiration and catheter-flushing techniques likely minimized these effects, confirming the safety of taurolidine–citrate as a lock solution [45].

Overall, our results partly contrast with the meta-analysis by Chapla et al. [46], which found no significant difference in catheter patency between heparin and alternative lock solutions. The totality of available evidence remains insufficient to support the routine use of taurolidine–citrate in pediatric oncology patients, though it appears safe and biochemically less pro-inflammatory.

This study was designed with catheter functional duration and IL-6 levels as the primary endpoints, while infection, thrombosis, and additional inflammatory markers were predefined as secondary, exploratory outcomes. Given the modest sample size and single time-point cytokine assessment, these inflammatory findings should be interpreted cautiously and validated through larger longitudinal studies.

## Limitations

Key limitations include the small sample size, heterogeneity of malignancy types, and open-label design, which may introduce performance and detection bias. Although laboratory analyses were blinded, clinicians responsible for catheter management were not. Additionally, the absence of serial inflammatory marker measurements and a saline-only control group limits interpretability. Future multicenter, double-blind, adequately powered trials with standardized outcome definitions and longer follow-up are warranted to clarify the clinical role of taurolidine–citrate in pediatric oncology patients. While references to adult and hemodialysis studies were included to provide mechanistic context, their clinical relevance to pediatric oncology remains limited and should be interpreted with caution.

## Conclusion

In this preliminary study, taurolidine-citrate demonstrated a modest anti-inflammatory profile through reductions in hs-CRP and WBC counts, without translating into significant improvements in catheter-related clinical outcomes. No significant effect was observed on IL-6 levels, catheter survival, or complication rates. These findings suggest limited short-term clinical benefit in pediatric oncology patients, possibly due to their complex clinical background. Further large-scale, controlled trials are warranted to clarify its therapeutic role and long-term impact in this high-risk population.

## Data Availability

The datasets used and/or aematol during the current study available from the corresponding author on reasonable request.

## Abbreviations

CVCs: Central venous catheters
CBC: Complete blood count
hs-CRP: Highly sensitive C-reactive protein
IL-6: Interleukin-6
CRBSIs: Catheter-related bloodstream infections
N/S: Normal saline
ELISA: Enzyme-linked immunosorbent assay
URR: Urea reduction ratio
BFR: Blood flow rate
SD: Standard deviation
HR: Hazard ratio
OR: Odds ratio
CI: Confidence interval
ALL: Acute lymphoblastic leukemia
AML: Acute myeloid leukemia
PNET: Pancreatic neuroendocrine tumors
LCH: Langerhans cell histiocytosis
ESRD: End-stage renal disease
WBC: White blood cell

## Declarations

### Transparency statement

The lead author Aziz Eghbali, Kazem ghaffari affirms that this manuscript is an honest, accurate, and transparent account of the study being reported; that no important aspects of the study have been omitted; and that any discrepancies from the study as planned (and, if relevant, registered) have been explained.
